# Radical surgical resection of advanced thymoma and thymic carcinoma infiltrating the heart or great vessels with cardiopulmonary bypass support

**DOI:** 10.1186/s13019-015-0346-2

**Published:** 2015-10-29

**Authors:** Michael Ried, Reiner Neu, Berthold Schalke, Marietta von Süßkind-Schwendi, Zsolt Sziklavari, Hans-Stefan Hofmann

**Affiliations:** 1 Department of Thoracic Surgery, University Medical Center Regensburg, Franz-Josef-Strauß-Allee 11, D-93053 Regensburg, Germany; 2 Department of Neurology, University Regensburg at the District Medical Center, Regensburg, Germany; 3 Department of Thoracic Surgery, Hospital Barmherzige Brüder Regensburg, Regensburg, Germany

**Keywords:** Thymoma, Thymic carcinoma, Cardiopulmonary bypass support, Radical surgical resection

## Abstract

**Background:**

Radical surgical resection of advanced thymic tumors invading either the heart or great vessels facing towards the heart is uncommonly performed because of the potential morbidity and mortality. To achieve a complete tumor resection, the use of cardiolpulmonary bypass (CPB) support might be necessary.

**Methods:**

Retrospective analysis of the results in six patients, who underwent radical tumor resection with CBP support.

**Results:**

Mean age was 46 years (27 to 66 years) and five patients were male. Tumor infiltration of the heart or the great vessels was evident in all patients. Five patients underwent induction therapy. Two patients were operated in complete cardioplegic arrest (antegrade cerebral perfusion: *n* = 1). Arterial cannulation of the ascending aorta (*n* = 5) or the femoral artery (*n* = 1) and venous cannulation of the right atrium (*n* = 4) or the femoral vein (*n* = 2) were performed. Resection of the left brachiocephalic vein (*n* = 6), resection of the superior caval vein (*n* = 2), the ascending aorta (*n* = 1) and the complete aortic arch with outgoing branches (*n* = 1) were performed. A macroscopic complete resection (R0/R1) was achieved in five patients, whereas one patient was resected incompletely (R2). In-hospital mortality was 0 %. Three (50 %) patients needed operative revision (hematothorax: *n* = 2, chylothorax: *n* = 1). All patients had a complicated postoperative course and developed respiratory insufficiency.

**Conclusions:**

Locally advanced thymoma/thymic carcinoma invading the heart or great vessels can be treated with radical surgical resection alongside with increased perioperative morbidity. The usage of CBP improves the chance of complete tumor resection in selected patients and might lead to a prolonged survival.

## Background

Complete surgical resection still builds the mainstay of treatment in all stages of thymoma and thymic carcinoma [1, 2]. Its prognosis depends on the preoperative tumor stage, as classified by the Masaoka-Koga staging system describing the anatomical extend of the mediastinal tumor, the World Health Organization (WHO) histological classification system and the completeness of surgical resection [3–5]. In particular, in Masaoka-Koga stage III tumors, defined by the macroscopic infiltration of neighbouring structures (i.e., mediastinal pleura, pericardium, lung tissue, and great vessels), and in stage IVa thymic tumors with pleural thymoma spread, a complete surgical resection is not always achievable [6–8]. A multimodality treatment regime consisting of chemotherapy, surgical resection and in some cases radiotherapy is recommended for advanced or primarily unresectable tumors [9, 10].

However, in this rare surgical condition no common and standardized treatment regime exists yet and the optimal way of therapy remains controversial [11, 12]. Most patients presenting with thymoma/thymic carcinoma invading the heart or great vessels are not considered for surgical resection because of technical reasons, the potential perioperative morbidity and mortality, and the unknown probability of significant impact on survival [13]. Therefore, these operative procedures are uncommonly performed and there have been only some case reports of patients, who underwent extended surgical resection with extracorporeal circulation in order to achieve complete tumor resection [14–17]. In this report we describe our experience of radical surgical tumor resection with cardiopulmonary bypass (CPB) support in patients with locally advanced thymoma or thymic carcinoma invading either the heart or great vessels facing towards the heart.

## Methods

This was an observational study. We reviewed the records of patients with advanced thymoma or thymic carcinoma, who received surgical resection with CPB support at the Department of Thoracic Surgery, University Medical Center Regensburg. Between November 2010 and October 2014 a total of six consecutive patients with locally advanced thymoma or thymic carcinoma Masaoka-Koga stage III and IVa with preoperative or intraoperative evidence of tumor infiltration of the heart, great vessels, or both, were included. Our Institutional Review Board waived the necessity of approval for the data report, because of using only routine patient data. All patients signed an informed consent form. Perioperative data and operative reports were obtained from the institutional database and medical records. Patients were recruited in an interdisciplinary thoracic oncology assessment involving thoracic surgeons, neurologists, oncologists, pulmonary specialists, radio-oncologists and radiologists. All perioperative complications regarding postoperative morbidity and mortality were documented.

All patients received a detailed preoperative history, physical examination, lung function testing and echocardiography to ensure functional operability. Preoperative staging included computed tomography (CT) scan of the chest in all patients. Cine magnetic resonance imaging (MRI) was performed to evaluate the extent of myocardial or great vessel invasion. The preoperative clinical and pathologic stage at the time of diagnosis was determined according Masaoka-Koga staging system [4, 18]. The pathologic results were classified according to the WHO histological classification system [5, 19].

Macroscopic complete surgical resection in a multimodality treatment setting was the aim in all patients. Induction therapy (chemotherapy or octreotid/prednisone) was administered in patients with large tumors, which were considered not completely resectable and in order to improve surgical resectability (partial remission: tumor reduction ≥50 %). Second-line treatments were administered when appropriate depending on tumor stage, histology and completeness of resection. In addition, adjuvant therapies were accomplished depending on the postoperative patient’s status and significant postoperative comorbidity. A further reason for not performing adjuvant therapy was patient refusal.

## Results

### Patient characteristics

**Fig. 1:**
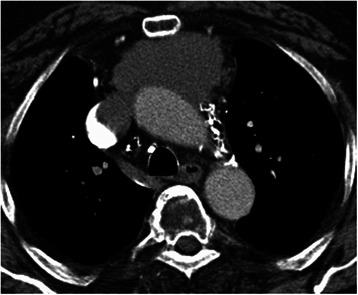
Thymoma Masaoka-stage III (WHO A) with infiltration of SCV and the right atrium with obstruction. Complete macroscopic resection (R0) with resection of the SCV and reconstruction with Dacron-prosthesis under CPB support (cannulation of the ascending aorta and the femoral vein)

**Fig. 2:**
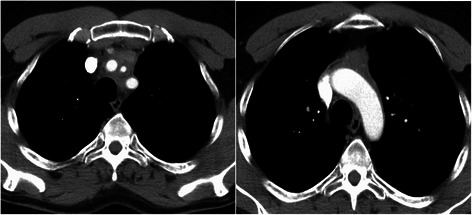
CT-scan of patient no. 5 with thymic carcinoma (WHO C) encircling the supraaortic branches and with suspicious invasion of the aortic arch

Patient characteristics and perioperative data are listed in Table 1. The study comprised six patients who underwent surgical resection with CPB support of an invasive thymoma or thymic carcinoma that invaded the myocardium, the superior caval vein (SCV), the left brachiocephalic vein (LBV), the ascending aorta and/or the aortic arch (Figs. 1 and 2). Included were five men and one woman with a mean age at time of surgery of 46 years (range 27 to 66 years). There were two patients with proven myasthenia gravis with one being without symptoms since one year before the re-operation due to suspicious mediastinal recurrence of thymoma in a time interval of 26 months after previous transsternal radical thymoma (WHO type B2) resection.

**Table 1:** Demographic and perioperative data

Pt	Age [years]/Sex/MG	Masaoka-Koga [3, 4]/WHO [5]	Induction therapy	Cannulation	Surgical approach: structures resected	CPB-/OP-time [min]	ICU-stay [days]
1	66/m/none	III/A	Octreotid/prednisone	Ascending aorta/femoral vein	Sternotomy: SCV, LBV, pericardium	94/269	28
2	27/m/none	IVa (left)/B3	PAC; octreotid/prednisone	Ascending aorta/RA	Sternotomy, left hemi clamshell thoracotomy: LBV, pericardium, left pleurectomy, left upper lobe	152/539	5
3	61/m/none	III/C	PAC	Femoral artery/femoral vein	Sternotomy: Chest wall, left upper lobe, pericardium, LBV, tumor debulking aortic arch and main pulmonary artery	378/664	28
4	35/f/MG	IVa (right)/B3	PAC; octreotid/prednisone	Ascending aorta/RA	Sternotomy, right hemi clamshell thoracotomy: EPP, pericardium, LBV	177/393	13
5	48/m/none	III/C	PAC	Ascending aorta/RA (hypothermic circulatory arrest)	Sternotomy: LBV, pericardium, aortic arch, brachiocephalic trunk, left carotid artery, left subclavian artery, wedge resections right and left lungs	240 (42)/467	26
6	41/m/MG	III/B2	None	Ascending aorta/RA	Sternotomy: LBV, ascending aorta	121/265	15

*CPB* cardiopulmonary bypass, *EPP* extrapleural pneumonectomy, *f* female, *LBV* left brachiocephalic vein, *m* male, *MG* myasthenia gravis, *OP* operation, *PAC* cisplatin, doxorubicin, cyclophosphamid, *Pt* patient, *RA* right atrium, *SCV* superior caval vein, *WHO* World Health Organication

After clinical and radiological staging the other five (83 %) patients with first diagnosis of thymoma/thymic carcinoma received induction therapy with chemotherapy or octreotid/prednisone. Partial remission was documented in all patients and surgical resection was the next step as part of the multimodality treatment concept. Curative resection was the intention to treat in all patients except one patient with expected tumor debulking surgery because of suspicious thymoma invasion in the myocardium of the left ventricle and the main pulmonary artery.

### Operative data

CPB was necessary in all patients to ensure a complete tumor resection as possible, to relieve the heart for better operability and to stabilize the hemodynamics during preparation. Furthermore, the decision for CPB support was made intraoperatively for safety reasons and for better tumor preparation. Except for one patient (femoral artery), the ascending aorta was cannulated. The venous cannulation was performed either in the right atrium (*n* = 4) or via the femoral vein (*n* = 2), depending on the central tumor invasion in the SCV or the right atrium. Heparin (350 IE/kg) was administered and CPB was performed with mild to moderate hypothermia (34 to 28 °C), except for one patient in whom hypothermic circulatory arrest (18 °C) was required for complete reconstruction of the aortic arch. Cardioplegic cardiac arrest was necessary in two patients who underwent aortic surgery.

**Fig. 3:**
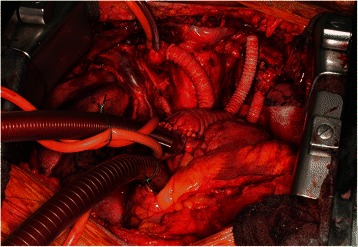
Intraoperative view (patient no. 5) after radical resection of the thymic carcinoma and vascular reconstruction of the aortic arch and the outgoing branches

The surgical procedure was preferably a radical tumor resection in all patients including the en bloc resection of the thymoma, along with the thymic gland, perithymic fat tissue, mediastinal pleura, and pericardium. When appropriate, the resection was extended to the lungs, phrenic nerves (unilateral) and the chest wall. In two patients with additional pleural tumor involvement left-sided radical pleurectomy with resection of the upper lobe or right-sided extrapleural pneumonectomy due to distinctive pulmonary tumor infiltration were performed. Reconstructions of the pericardium or the diaphragm were performed with bovine patches. The resected SCV was replaced by a ring-augmented PTFE-prosthesis (Polytetrafluorethylene; *n* = 1), whereas the LBV was either replaced (PTFE-prosthesis: *n* = 3) or closed (*n* = 3). One patient with suspicious mediastinal thymoma recurrence underwent re-sternotomy and showed intraoperative tumor infiltration in the ascending aorta, which was resected and reconstructed with a dacron-prosthesis (Hemashield vascular prosthesis 28 mm). Final histology ensured only scarred tissue without evidence of recurrent thymoma. A second patient had an invasive thymic carcinoma invading the aortic arch with complete immured supraaortic branches (Fig. 2). The aortic arch was replaced with a Hemashield vascular prosthesis (22 mm) under hypothermic circulatory arrest (42 min) with selective, antegrade cerebral perfusion. Additionally, the brachiocephalic trunk (8 mm), the left carotid artery (6 mm) and the left subclavian artery (6 mm) were reconstructed with vascular prostheses and connected to the aortic arch prosthesis (Fig. 3). Histological examination confirmed a thymic carcinoma (WHO type C) with invasion into the aortic wall, the brachiocephalic trunk, left carotid artery and the left subclavian artery, which has been completely resected (R0).

A complete tumor resection (R0) was achieved in four (66.6 %) patients. A microscopic or macroscopic incomplete tumor resection was performed in two patients, due to tumor infiltration of the aortic arch (R1: *n* = 1) or the aortic arch, the main pulmonary artery and the myocardium of the left ventricle (R2: *n* = 1).

### Postoperative data

Postoperative data are shown in Table 2. There was no in-hospital mortality or death within 30 days after operation in this series. Severe postoperative complications requiring soon operative revision were observed in half of the patients (50 %). Two patients with a hematothorax underwent successful operative revision. On the third postoperative day, in one patient after extended reconstruction of the aortic arch left-sided chylothorax was diagnosed and the patient underwent successful surgical revision. The postoperative course was prolonged and complicated by severe pneumonia with respiratory insufficiency and the need for tracheotomy. The patient has been discharged in a peripheral hospital 47 days after surgery. We disclaimed postoperative mediastinal radiation therapy because of the reduced overall condition of the patient.

**Table 2:** Postoperative and follow-up data

Pt	T [20]/UICC	Extent of resection/Residual disease	Postoperative complications	Progress/Recurrence [months]	Status/Survival^a^ [months]
1	3/IIIa	R0/none	Apoplex, respiratory insufficiency, tracheotomy	None	NED/55
2	4/IIIb	R1/aortic arch	Pneumonia, respiratory insufficiency, wound healing disorder	Thymoma metastasis above left diaphragm, pleural empyema: complete resection (R0), decortication	NED/20
3	4/IIIb	R2/aortic arch, main pulmonary artery, myocardium	Pneumonia, respiratory insufficiency, tracheotomy	Mediastinal tumor progress	DOD/7
4	3/IIIa	R0/none	Pneumonia, respiratory insufficiency, tracheotomy	None	NED/25
5	4/IIIb	R0/none	Pneumonia, respiratory insufficiency, tracheotomy, gastrointestinal bleeding	None	NED/8
6	4/IIIb	R0/none	Pneumonia, respiratory insufficiency, myasthenic crisis	None	NED/8

*UICC* Union for International Cancer Control, *T* T-category, *R0* no residual tumor, *R1* microscopic residual tumor, *R2* macroscopic residual tumor, *CT* chemotherapy, *RT* radiotherapy, *NED* no evidence of disease, *DOD* dead of disease

^a^Follow-up: June 2015

Every patient developed a postoperative respiratory insufficiency with the need for reintubation with prolonged mechanical ventilation followed by tracheotomy (*n* = 5). Despite there was no evidence of recurrent thymoma and the patient has been free of symptoms of his myasthenia gravis over one year, the patient developed in the early postoperative period a symptomatic myasthenic crisis combined with a pronounced pneumonia of the left lower lobe. The patient could be successfully treated with intravenous pyridostigmin and non-invasive ventilation (*n* = 1). Postoperative renal insufficiency with temporary renal replacement therapy was necessary in three (50 %) patients. The median length of stay on the intensive care unit (ICU) was 26 days (range: 5 to 28 days). Except for one patient (discharge home from hospital after 31 days) all patients were directly transferred from the ICU to a peripheral hospital or rehabilitation clinic.

Adjuvant radiotherapy was administered in one patient with acceptable good postoperative clinical conditions after R1-resection. In the other patients no further adjuvant therapy was recommended because of their impaired clinical overall condition after a prolonged postoperative course. At the end of the study in June 2015, 83 % (5/6) patients were alive without evidence of recurrence after a mean follow-up of 20.5 months (range: 7 to 55 months). One patient died 7 months after surgery due to further tumor progress after an incomplete tumor resection (R2). The functional outcome of the patients was good.

## Discussion

The prognosis of thymoma and thymic carcinoma depends on resection status, tumor stage, until now classified by the Masaoka-Koga staging system which describes the anatomic invasion into surrounding mediastinal structures or the pleural cavity, and, at least in most studies, on WHO-based histology [3–5, 18, 19]. Recently, there is a proposed tumor, node, metastasis (TNM)-based system, which should be applicable to all types of thymic epithelial malignancies [20]. In addition, complete surgical resection is still the most important determinant of survival in these patients [2, 7, 21, 22]. Especially in patients with advanced thymoma, surgical resection should be part of a multimodality therapy including chemotherapy and in some cases adjuvant radiotherapy to decrease the risk of recurrence and improve survival [8, 9, 23–25].

Nevertheless, malignant thymic tumors invading the heart or great vessels are rarely considered for surgical resection with curative intention [13]. In the literature there is only limited data about radical surgical interventions with CPB support presenting only case reports or case series with small numbers of patients [14–17]. In our series we describe six patients, who underwent radical surgical tumor resection with CPB support due to tumor invasion into the heart or great vessels. All patients were carefully selected for this aggressive therapy after multidisciplinary evaluation and proven operability. Finally, this therapy should be at least considered in patients who have no further therapeutic alternatives and only in experienced thoracic surgical centres [13].

Induction therapy is recommended in locally advanced thymomas not eligible for immediate resection [10]. In particular, patients presenting with a large, locally advanced thymoma that is likely to invade adjacent intrathoracic organs, induction chemotherapy is utilized to increase resectability with an objective response rate of approximately 50 % after platin-based combination chemotherapy [6]. Five (83 %) of our patients received induction chemotherapy with according to the PAC regime or, if octreotid-positive, with octreotid/prednisone to ensure better resectability. In all five patients a partial remission (positive response rate) was observed after reevaluation with computed tomography scans. Although, we only included locally advanced mediastinal tumors with potential invasion of the heart or great vessels, our rate of a macroscopic complete resection with removal of all visible tumor masses was approximately 83 %. Tumor debulking surgery (R2) was performed in one patient with massive thymic carcinoma invasion. In our experience and to our knowledge in the literature, no curative, complete resection of invasive thymic malignancies is possible, if there is preoperative suspicion or intraoperative evidence of tumor infiltration in the myocardium of the right/left ventricle. However, this situation should not primarly be a contraindication for surgery, because tumor debulking surgery combined with chemotherapy and radiotherapy is known to improve survival in these patients [1, 12].

Great vessel reconstruction can be performed with prosthetic material if there is an extensive tumor invasion of the chest wall [13]. The prosthetic graft patency rates in the left innominate vein position were disappointing (0 %) and in the future we might just ligate the vein. Complete reconstruction of the aortic arch under antegrade cerebral perfusion was performed in one patient (male, 48 years) with an invasive thymic carcinoma as previously described by Fujino et al. [14].

All of our patients (100 %) developed postoperative respiratory insufficiency related to pneumonia and also suffered from a systemic inflammatory response syndrome (SIRS). These complications might be triggered by the usage of the CPB in tumor patients with already preoperative immunodeficiency related to their malignant disease followed by induction chemotherapy and extended surgical resections of their malignant tumors. In addition to the postoperative intensive care treatment, these patients might benefit from prophylactic, perioperative intravenous antibiotic therapy and additional treatment with immunoglobulines, when appropriate. Furthermore, thymoma patients could develop postoperative paraneoplastic syndromes including myasthenic crisis irrespective of their preoperative neurological condition and medication.

Our current overall calculated median survival rate at the end of the study period in June 2015 was 23 months with five patients being alive without evidence of disease or tumor recurrence. In our department, adjuvant radiotherapy is used in patients with incomplete macroscopic (R2) or microscopic (R1) resection and patients with higher histologic thymoma stages or thymic carcinoma. In this series, only one patient developed adjuvant radiotherapy.

Finally, to our knowledge and experience, the risk of systemic dissemination of tumor cells during CPB seems to be acceptable and lower compared to the risk of haematogenic tumor cell dissemination caused by the vessels invasion of the malignant thymoma or thymic carcinoma.

We are aware of the potential restrictions of our study. The obvious limitation is its observational nature with only a small sample size including six patients without any control group. Nevertheless, we could demonstrate representative patients with large invasive thymoma or thymic carcinoma who underwent radical surgical tumor resection under CPB support.

## Conclusions

In conclusion, extensive surgical resections of advanced thymoma/thymic carcinoma infiltrating the heart or great vessels with CPB support are technical feasible in selected patients, but with a significant increased postoperative morbidity. Great vessel infiltration or infiltration of the right atrium should not primarily be a contraindication for surgery. CPB support enables complete tumor resection in selected patients and might lead to a better palliation and a potential for cure with prolonged survival. Surgical resection should always be performed as part of a multimodality therapy in this selected group of patients and only in experienced thoracic surgical departments.
